# Neural mechanisms of symmetry perception: hemispheric specialization and the impact of noise on reflection symmetry detection

**DOI:** 10.3389/fnins.2025.1599112

**Published:** 2025-05-14

**Authors:** Meng Wang, Jingjing Yang, Yiyang Yu, Qiong Wu, Fengxia Wu

**Affiliations:** ^1^School of Artificial Intelligence, Changchun University of Science and Technology, Changchun, China; ^2^Laboratory of Psychological Testing and Behavior Analysis, Liaoning University, Shenyang, Liaoning, China; ^3^Department of Psychology, Suzhou University of Science and Technology, Suzhou, China

**Keywords:** symmetry perception, hemispheric specialization, EEG, neural mechanisms, event-related potentials (ERPs)

## Abstract

Symmetry is a crucial cue for perceptual grouping in human vision. This study investigates the neural and cognitive mechanisms underlying symmetry perception, focusing on hemispheric specialization and the effects of noise on symmetry detection. Using psychophysical and electrophysiological (EEG) experiments, participants were presented with reflection symmetric patterns (full circle vs. right-left quarter-circle), under varying noise levels. Behavioral results demonstrated noise-induced impairment in accuracy (*p* < 0.001), with Cycle outperforming Quarter in noiseless conditions (*p* < 0.05), highlighting the role of contour completeness in perceptual grouping. EEG recordings revealed distinct neural mechanisms associated with different stages of symmetry processing. Early sensory processing exhibited left-hemisphere dominance, while later stages implicated the right hemisphere in noise-modulated global integration. Noise disrupted early contour integration and attenuated higher-order object recognition processes, with right-hemisphere sensitivity to noise emerging during decision-making. These findings challenge the strong version of the callosal hypothesis, highlighting the complexity of hemispheric interactions in symmetry perception. This study provides new insights into the interplay between bottom-up sensory processing and top-down hemispheric interactions in perceptual organization.

## 1 Introduction

The human visual system is remarkably adept at organizing fragmented or incomplete visual input into coherent perceptual objects. This ability is governed by the principles of perceptual grouping, as outlined by the Gestalt laws of perception, which include principles such as good continuation, closure, proximity, similarity, and symmetry (Gorbunova, 2017; Mauro and David, 2024; Purves, 2024; Wang et al., 2022). Among these, symmetry has been identified as a particularly salient cue for perceptual grouping (Moscoso et al., 2023; Henle, 1963; Hu et al., 2024; Purves, 2024). Symmetry allows the visual system to efficiently segregate objects from their backgrounds and to infer the structure of partially occluded or ambiguous stimuli (Moscoso et al., 2022). Ernst Mach (Mach, 1914) was among the first to systematically categorize symmetry into three types: translational (repetition) symmetry, reflectional (mirror) symmetry, and centric (rotational) symmetry. Of these, reflectional symmetry, particularly mirror symmetry, has been shown to be processed more readily and rapidly than other forms of symmetry or asymmetrical patterns (Palmer and Hemenway, 1978; Wright, 1972). This efficiency in processing mirror symmetry is thought to play a critical role in the perceptual organization of visual scenes, helping to group and segregate visual input into meaningful objects and backgrounds (Machilsen et al., 2009).

### 1.1 Neural mechanisms of symmetry perception

Recent advances in neuroimaging and electrophysiological techniques have provided new insights into the neural mechanisms underlying symmetry perception (Beck et al., 2005; Carr et al., 2025). Functional magnetic resonance imaging (fMRI) and electroencephalography (EEG) studies have identified a distributed network of brain regions involved in processing symmetrical patterns. These include early visual areas such as the primary visual cortex (V1) and extrastriate areas (V2, V4), as well as higher-order regions like the lateral occipital complex (LOC) and the intraparietal sulcus (IPS) (Bertamini et al., 2018; Makin et al., 2021). These regions work in concert to encode local symmetrical features and integrate them into global perceptual representations. For example, Tyler et al. (2005) used fMRI to investigate the LOC exhibits strong neural responses to symmetry patterns (such as mirror and rotational symmetry), supporting its role in symmetry grouping and perception (Tyler et al., 2005). This suggests that the LOC plays a critical role in the perceptual grouping of symmetrical patterns. Similarly, Bertamini et al. (2018) demonstrated that symmetry processing involves both feedforward and feedback mechanisms, with early visual areas encoding local features and higher-order regions integrating these features into coherent perceptual objects (Bertamini et al., 2018).

### 1.2 Cognitive and contextual influences on symmetry perception

While neural mechanisms provide the foundation for symmetry detection, cognitive factors such as attention, expectation, and learning also play a significant role (Elena et al., 2021; Makin et al., 2023). Recent studies have shown that symmetry perception can be modulated by top-down processes, such as attentional focus and task demands. For instance, research by Bertamini and Makin (2014) demonstrated that attention can enhance the perception of symmetry, particularly when participants are explicitly instructed to focus on symmetrical patterns (Bertamini and Makin, 2014). This suggests that attention can amplify the neural responses to symmetry, making it more salient in the visual field. Moreover, understanding hemispheric specialization in symmetry perception is critical for cognitive neuroscience because it reveals fundamental principles of how the brain achieves perceptual organization (Rabbito et al., 2023). The two hemispheres exhibit distinct processing biases - with the left hemisphere preferentially analyzing local features and the right hemisphere specializing in global integration (Fink et al., 1997; Makin et al., 2021; Paulraj et al., 2018). As symmetry detection requires both local element processing and global configuration analysis, it provides an ideal paradigm to investigate how divided hemispheric computations are coordinated through callosal connections.

In this study, we aim to further explore the mechanisms underlying symmetry perception, with a particular focus on the role of the two cortical hemispheres in the detection of reflection symmetric patterns. Specifically, does there exist hemisphere specialization for the detection of reflection symmetric patterns? The callosal hypothesis posits that the anatomical symmetry of the human visual system underlies the efficiency of vertical symmetry detection. However, recent evidence suggests that symmetry perception may involve more complex interactions between the two hemispheres. We aim to examine whether there is a functional specialization of the left and right hemispheres for processing different orientations of reflection symmetry.

To address these issues, we conducted a series of psychophysical and electrophysiological experiments in which participants were presented with two types of symmetrical patterns: cycle and right-left quarter-circle. These patterns were presented in the right, left, or both visual fields to assess the role of hemispheric processing in symmetry detection. By combining behavioral measures with EEG recordings, we aim to provide a comprehensive understanding of the neural and cognitive mechanisms underlying symmetry perception.

## 2 Materials and methods

### 2.1 Participants

Fifteen students (10 males; mean age = 26.3; SD = 3.4) with normal or corrected-to-normal vision from Okayama University volunteered for the experiments. They provided written informed consent for their participation in this study, which was previously approved by the ethics committee of Okayama University.

### 2.2 Apparatus and stimuli

**FIGURE 1:**
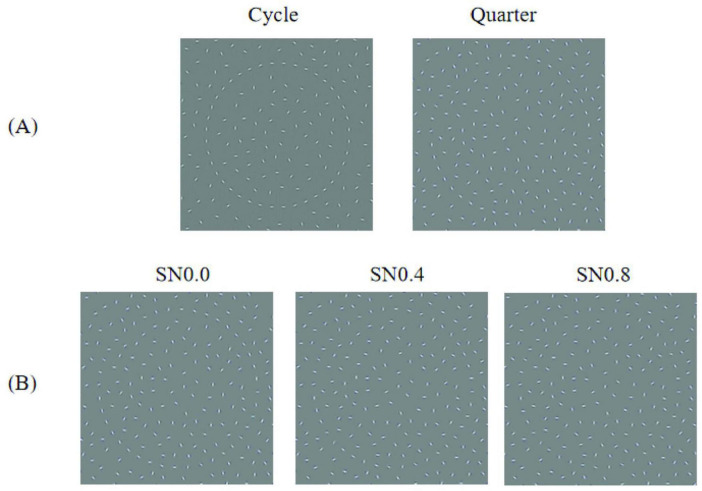
Base stimulus used in the experiment. **(A)** Examples of two types of stimuli. **(B)** Examples of three different noise levels.

We used MATLAB (v 14; the MathWorks, Okayama, Japan) and GERT, the Grouping Elements Rendering Toolbox (Demeyer and Machilsen, 2012), to construct arrays of non-overlapping Gabor elements on a uniform gray background (Figure 1). The arrays comprised 496 × 496 pixels. Each Gabor element was defined as the product of a sine wave luminance grating (frequency of 3 cycles per degree of visual angle and presented at 100% Michelson contrast) and a circular Gaussian (standard deviation of 3 arc min). There were 512 Gabor elements, interior 62 elements, exterior 410 elements and outline 40 elements. The number of elements inside and outside the shape outline was adjusted to ensure a homogeneous spacing between the Gabor elements. The orientations of the inside and outside Gabors were randomly and unchanged. A subset of 40 Gabor elements were positioned along the contour outline of an artificial circle shape. The shape outline was generated by plotting the sum of 5 radial frequency components in polar coordinates. The orientation of Gabor on contour was defined by stimulus type and stimulus intensity. There were two kinds of stimulus type: circle, right-left quarter-circle, see Figure 1A. The elements had orientations parallel to the local tangent of the shape outline. For example, the orientation of left 20 Gabors were local tangent of the shape outline but right 20 Gabors were randomly when stimulus was left semicircle. In addition, we created three different noise levels by altering the orientations of the contour elements. As shown in Figure 1B, Stimuli noise 0.0 (SN0.0): The orientations of the Gabors are aligned with the local tangents of the shape contours; Stimuli noise 0.4 (SN0.4): Randomly change the orientations of the 8 Gabors; Stimuli noise 0.8 (SN0.8): Randomly change the orientations of the16 Gabors.

### 2.3 Procedure and task

**FIGURE 2:**
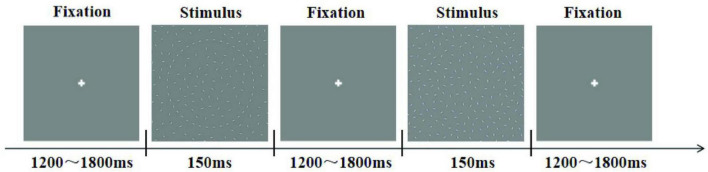
Trial structure of the experiment.

The subjects were instructed to perform the experiment in a dimly lit, electrically shielded and sound-attenuated room (laboratory room, Okayama University, Japan) with their head positioned on a chin rest. Stimulus presentation and response collection were conducted using E-prime 1.1 software (Psychology Software Tolls, Inc., Pittsburgh, PA, USA). Figure 2 showed the experiment consisted of three sessions of 120 trials each, all stimuli were presented randomly. For each stimulus condition, subjects were presented with a central fixation cross for a randomized duration between 1200 and 1800 ms, followed by the stimulus display for 150 ms. During the experiment, participants were instructed to press the “1” key on the keyboard when a “Cycle” stimulus was displayed, the “2” key for a “left quarter-circle” stimulus, and the “3” key for a “right quarter-circle” stimulus.

### 2.4 EEG recording and analyses

An EEG system (BrainAmp MR plus, Gilching, Germany) was used to record EEG signals through 32 electrodes mounted on an electrode cap (Easy Cap, Herrsching-Breitbrunn, Germany). Horizontal eye movements were measured by deriving the electrooculogram (EOG) from one electrode placed about 1cm from the outer canthi of the left eye. Vertical eye movements and eye blinks were detected by deriving an EOG from an electrode placed approximately 1.5 cm below the subject’s left eye. All signals were referenced to left and right earlobe, and the impedance was maintained below 5 kΩ. Raw signals were acquired at a sample rate of 500 Hz and stored for off-line analysis.

The ERPs elicited by target stimuli were analyzed by using the Brain Vision Analyzer software (version 1.05, Brain Products GmbH, Munich, Germany). The data were band-pass filtered from 0.01 to 30 Hz. Then, the data were divided into epochs, from −100 ms before stimulus onset to 500 ms after stimulus onset, and baseline corrections were made to the data from −100 ms to stimulus onset. Epochs contaminated by artifacts (i.e., eye movements, eye blinks, amplifier blocking) were rejected based on a threshold of ±100 μV in all channels before averaging. All averaged ERP waveforms were obtained across all participants for each stimulus type in each electrode.

**FIGURE 3:**
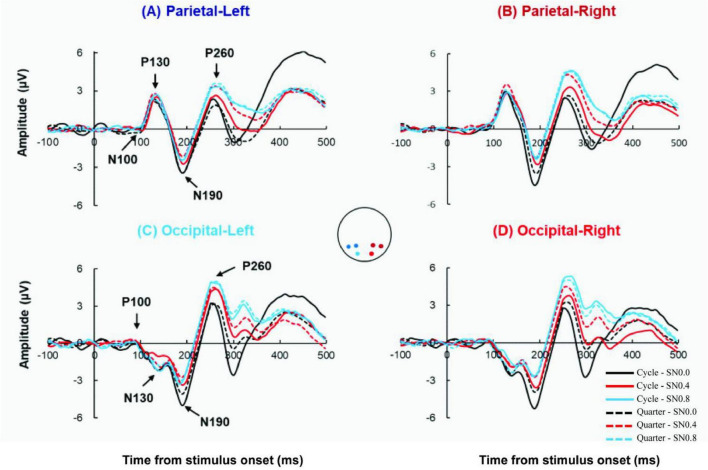
Grand-average ERP wave from 4 ROIs. **(A)** The Grand-average ERP waves from left parietal with 2 stimulus types (cycle: solid line; quarter: dot line) in SN0.0, SN0.4, SN0.8. Equivalent data from right parietal **(B)**, left occipital **(C)** and right occipital **(D)**.

Therefore, our analysis was performed across electrode regions of interest focused at left occipital region (O1), right occipital region (O2), left parietal region (P3 and P7), right parietal region (P4 and P8), see Figure 3.

Murray et al. (2006) found that both correct and incorrect responses did not affect the contour integration effect or shape detection within the first 300 milliseconds (Murray et al., 2006). Therefore, four main components before 300 ms were statistically analyzed. The first component was the P/N100 complex (peaking around 100 ms after stimulus onset); the second component was the P/N130 complex (peaking around 130 ms after stimulus onset); the third component was the N190 (peaking around 190 ms after stimulus onset); the last component was P260 (peaking around 260 ms after stimulus onset). Amplitudes of these component respectively measured as an average of 20 ms duration around the peak. ANOVA (Greenhouse-Geisser corrections with corrected degrees of freedom).

## 3 Results

### 3.1 Behavioral results

Table 1 showed the proportion correct for the 6 stimuli. Analysis of the proportion correct using 2 (stimulus type) × 3 (noise level) repeated measures ANOVA revealed a significant main effect in noise level [F (2, 28) = 190.008, *p* < 0.001, η_*p*_^2^ = 0.931], showing that the identification ability of participants was decreased with noise enhancement. Whereas, no significant difference was found in stimulus type [F (1, 14) = 2.663, *p* = 0.125, η_*p*_^2^ = 0.160]. The interaction between stimulus type and noise level was significant [F (2, 28) = 6.705, *p* = 0.012, η_*p*_^2^ = 0.324], indicated that contour effect was different in varying noise level. The pairwise comparisons showed that for the SN 0.0 condition, the responses to cycle stimuli were significantly higher than the responses to quarter stimuli (*p* < 0.05). All reported statistics reflect Green-Geisser corrections at a significance level of 0.05.

**TABLE 1:** Behavior results of the proportion correct.

Conditions	SN0.0*	SN0.4	SN0.8
Cycle	0.89 ± 0.03	0.42 ± 0.06	0.01 ± 0.00
Quarter	0.78 ± 0.04	0.28 ± 0.06	0.08 ± 0.04

*Denotes statistical significance (*p* < 0.05).

### 3.2 ERP results

Figure 3 showed the ground-averaged ERPs across all participants for 6 stimuli. All stimuli evoked similar P100, N130, N190, P260 components at occipital electrode, and evoked N100, P130, N190, P260 components at parietal electrode. Analysis of the peak latency with 4 components revealed there was no significant difference in 6 stimuli across both hemispheres and electrodes (all *p* > 0.05). Analysis of the mean amplitude of 4 components (P/N100, P/N130, N190, P260) × 2 electrode (occipital, parietal) × 2 hemisphere (left, right) × 2 stimuli type (cycle, quarter) × 3 noise level (SN0.0, SN0.4, SN0.8) repeated measures ANOVA revealed a significant four-way component × electrode × hemisphere × stimuli type × noise level interaction [F (6, 84) = 3.937, *p* = 0.015, η_*p*_^2^ = 0.219]. The results suggested different processing patterns for different noise conditions and for left hemisphere and right hemisphere. Therefore, we analyzed these differences in detail as follows.

#### 3.2.1 P/N100 component

**FIGURE 4:**
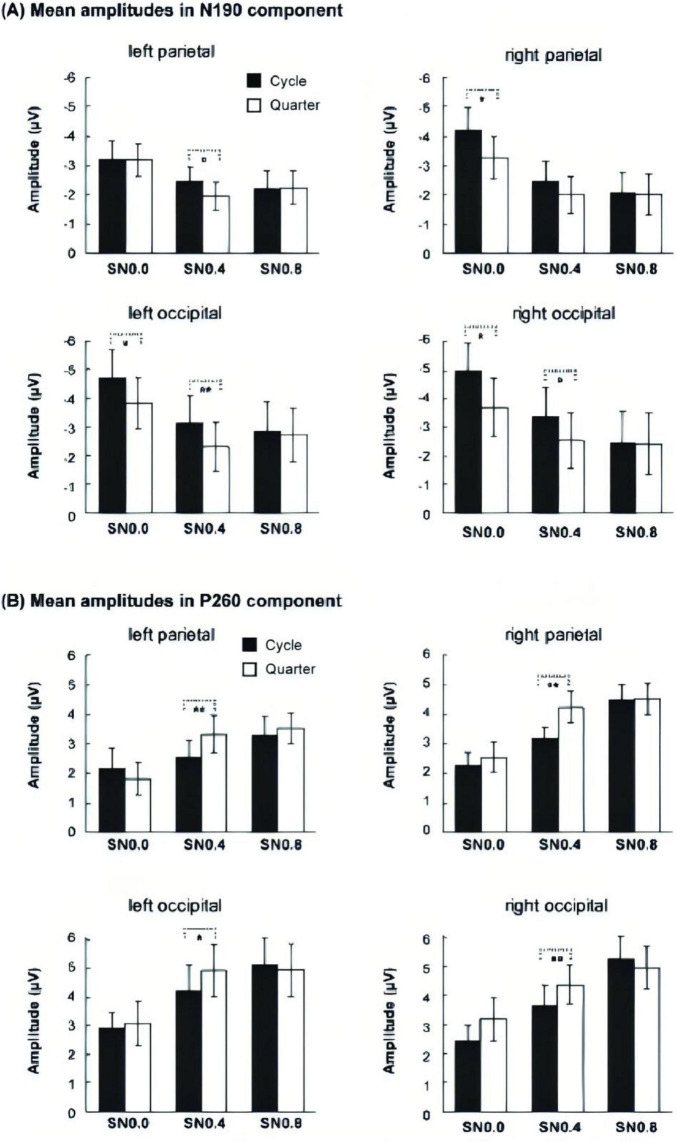
Mean amplitudes of three noise level across four components. **(A)** Mean amplitudes of P100 in occipital. **(B)** Mean amplitudes of N130 in occipital. **(C)** Mean amplitudes of N190 ms over four ROIs. **(D)** Mean amplitude of P260 over four ROIs. *p < 0.05; **p < 0.01; ***p < 0.001.

Apparently, for this component, there was a clear inversion across the electrode sites. Therefore, ANOVA tests were conducted separately for occipital and parietal electrode using the factors hemisphere, stimulus type and noise level. For the occipital electrode, the mean amplitude was submitted to a 2 hemisphere (left, right) × 2 stimuli type (cycle, quarter) × 3 noise level (SN0.0, SN0.4, SN0.8) ANOVA, no main effect or interaction were observed, see Figure 4A. Whereas, for the parietal electrode, a main effect of hemisphere was found [F (1, 14) = 8.056, *p* = 0.013, η_*p*_^2^ = 0.365], with a more negative going response to left hemisphere than right hemisphere.

#### 3.2.2 P/N130 component

Similar with P/N100 component, ANOVA tests in P/N130 component were conducted separately for occipital and parietal electrodes. The 2 hemispheres (left, right) × 2 stimuli type (cycle, quarter) × 3 noise level (SN0.0, SN0.4, SN0.8) ANOVA for occipital electrode revealed a main effect of noise level [F (2, 28) = 6.896, *p* = 0.005, η_*p*_^2^ = 0.33], with N130 being less negative for noise increasing, see Figure 4B. Further *post hoc* analysis revealed a significant difference between SN0.0 and SN0.4 (*p* < 0.05), a marginally difference between SN0.0 and SN0.8 (*p* = 0.069), while the difference between SN0.4 and SN0.8 was not significant (*p* > 0.05). The 2 hemispheres (left, right) × 2 stimuli type (cycle, quarter) × 3 noise level (SN0.0, SN0.4, SN0.8) ANOVA for parietal electrode revealed that there were no main effect or interaction were observed (all *p* > 0.05).

#### 3.2.3 N190 component

**FIGURE 5:**
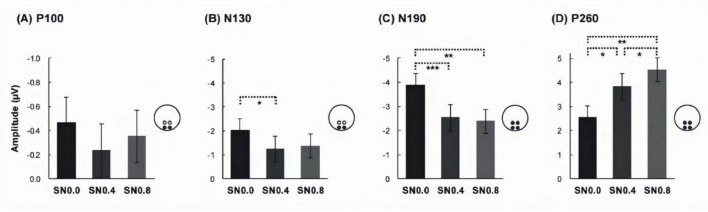
Mean amplitudes of two stimulus types across three noise level at **(A)** N190 component and **(B)** P260 component. *p < 0.05, **p < 0.01.

For the average amplitude of N190 component, 2 electrodes (occipital, parietal) × 2 hemisphere (left, right) × 2 stimuli type (cycle, quarter) × 3 noise level (SN0.0, SN0.4, SN0.8) ANOVA revealed a significant main effect of stimuli type [F (1, 14) = 9.778, *p* = 0.007, η_*p*_^2^ = 0.411], noise level [F (2, 28) = 32.26, *p* < 0.001, η_*p*_^2^ = 0.697]. The following two-way and three-way interactions were significant: electrode × stimuli type [F (1, 14) = 5.957, *p* = 0.029, η_*p*_^2^ = 0.299], hemisphere × noise level [F (2, 28) = 4.439, *p* = 0.038, η_*p*_^2^ = 0.241], electrode × stimuli type × noise level [F (2, 28) = 3.936, *p* = 0.037, η_*p*_^2^ = 0.219], hemisphere × stimuli type × noise level [F (2, 28) = 4.962, *p* = 0.016, η_*p*_^2^ = 0.262]. The *post hoc* analysis for stimulus type on each noise level and ROI showed that the amplitude of cycle stimulus in SN0.0 and SN0.4 was significantly more negative than that for quarter stimulus at left and right occipital (all *p* < 0.05). Whereas, this difference in parietal was only found in right hemisphere at SN0.0 (*p* = 0.032) and left parietal at SN0.4 (*p* = 0.030). However, no significant difference was found in SN0.8, see Figure 5. In addition, the *post hoc* analysis for noise level on both stimulus type revealed a significant difference between SN0.0 and SN0.4 (*p* < 0.001), between SN0.0 and SN0.8 (*p* < 0.01), while the difference between SN0.4 and SN0.8 was not significant (*p* > 0.05) in all four ROIs, see Figure 4C.

#### 3.2.4 P260 component

For the average amplitude of P260 component, the 2 electrodes (occipital, parietal) × 2 hemisphere (left, right) × 2 stimuli type (cycle, quarter) × 3 noise level (SN0.0, SN0.4, SN0.8) ANOVA revealed a significant main effect of stimuli type [F (1, 14) = 6.569, *p* = 0.023, η*_*p*_*^2^ = 0.319], noise level [F (2, 28) = 32.679, *p* < 0.001, η*_*p*_^2^* = 0.70]. In addition, three-way interactions of electrode × stimuli type × noise level [F (2, 28) = 6.417, *p* = 0.01, η_*p*_^2^ = 0.314], and hemisphere × stimuli type × noise level [F (2, 28) = 4.218, *p* = 0.028, η*_*p*_^2^* = 0.232] were also significant. The *post hoc* analysis for stimulus type on noise level and ROI showed that the amplitude of cycle stimulus in SN0.4 was significantly less positive than that for quarter stimulus at four ROIs (all *p* < 0.05). In addition, further *post hoc* analysis for noise level on both cycle and quarter stimulus revealed a significant difference between SN0.0 and SN0.4 (*p* < 0.05), between SN0.0 and SN0.8 (*p* < 0.05), and the difference between SN0.4 and SN0.8 was also significant (*p* < 0.01) in all four ROIs, see Figure 4D.

## 4 Discussion

The present study aimed to investigate the neural and cognitive mechanisms underlying the perception of reflection symmetry, with a particular focus on the role of hemispheric specialization and the effects of noise on symmetry detection. By combining behavioral measures with high-density EEG recordings, we explored how different types of symmetrical patterns (full circle vs. quarter circle) and varying noise levels influence the efficiency of symmetry perception. Our findings provide new insights into the temporal dynamics and neural correlates of symmetry processing, as well as the interplay between bottom-up and top-down mechanisms in visual perception.

The behavioral results revealed that participants’ ability to identify symmetrical patterns decreased significantly as noise levels increased, consistent with previous studies demonstrating that noise disrupts contour integration and perceptual grouping (Field, 1992; Hess and Field, 1999; Baldwin et al., 2017). Interestingly, while there was no overall difference in accuracy between full circle and quarter circle stimuli, a significant interaction between stimulus type and noise level indicated that the contour effect was modulated by noise. Specifically, in the absence of noise (SN0.0), participants performed significantly better with full circle stimuli compared to quarter circle stimuli. This suggests that the completeness of the symmetrical contour plays a critical role in facilitating perceptual grouping under optimal viewing conditions. However, as noise levels increased, this advantage diminished, highlighting the vulnerability of contour integration to external noise. As shown in Table 1, analysis of the proportion correct using a 2 (stimulus type) × 3 (noise level) repeated measures ANOVA revealed a significant main effect of noise level [F (2, 28) = 190.008, *p* < 0.001, ηp^2^ = 0.931], showing that the identification ability of participants decreased with noise enhancement. Whereas, no significant difference was found in stimulus type [F (1, 14) = 2.663, *p* = 0.125, ηp^2^ = 0.160]. The interaction between stimulus type and noise level was significant [F (2, 28) = 6.705, *p* = 0.012, ηp^2^ = 0.324], indicating that the contour effect was different under varying noise levels. The pairwise comparisons showed that for the SN0.0 condition, the responses to cycle stimuli were significantly higher than the responses to quarter stimuli (*p* < 0.05).

The ERP results provided a detailed temporal profile of symmetry processing, revealing distinct neural components associated with different stages of visual perception (see Table 2). The P/N100 complex, peaking around 100 ms after stimulus onset, is thought to reflect early sensory processing in the primary visual cortex (V1) and extrastriate areas (V2/V4) (Francesco et al., 2002). In our study, the P/N100 component showed no significant differences across stimulus types or noise levels at occipital electrodes, suggesting that early sensory encoding of symmetrical patterns is relatively robust to noise. However, at parietal electrodes, a significant hemisphere effect was observed, with more negative amplitudes in the left hemisphere compared to the right. This asymmetry may reflect differential engagement of the two hemispheres in early visual processing, consistent with previous findings of hemispheric specialization in contour integration (Kovács et al., 1999).

**TABLE 2:** Mean amplitudes of ERP components across noise levels.

Component	Electrode	Hemisphere	SN0.0	SN0.4	SN0.8
P/N100	Occipital	Left	−2.5 ± 0.3	−2.4 ± 0.3	−2.3 ± 0.3
		Right	−2.4 ± 0.3	−2.3 ± 0.3	−2.2 ± 0.3
	Parietal	Left	−3.1 ± 0.4	−3.0 ± 0.4	−2.9 ± 0.4
		Right	−2.8 ± 0.4	−2.7 ± 0.4	−2.6 ± 0.4
N130	Occipital	Left	−4.2 ± 0.5	−3.8 ± 0.5	−3.5 ± 0.5
		Right	−4.1 ± 0.5	−3.7 ± 0.5	−3.4 ± 0.5
N190	Occipital	Left	−5.6 ± 0.6	−4.8 ± 0.6	−4.0 ± 0.6
		Right	−5.5 ± 0.6	−4.7 ± 0.6	−3.9 ± 0.6
P260	Parietal	Left	3.2 ± 0.4	2.8 ± 0.4	2.4 ± 0.4
		Right	3.0 ± 0.4	2.6 ± 0.4	2.2 ± 0.4

The N130 component, peaking around 130 ms, is associated with the initial stages of contour integration and perceptual grouping (Murray et al., 2004). Our results showed that the N130 amplitude at occipital electrodes became less negative as noise levels increased, indicating that noise disrupts the early stages of contour integration. This finding aligns with the behavioral results, further supporting the idea that noise impairs the ability to group local elements into coherent global shapes.

The N190 component, peaking around 190 ms, is thought to reflect higher-order processing in the lateral occipital complex (LOC) and other extrastriate areas involved in object recognition (Kourtzi and Kanwisher, 2001). In our study, the N190 amplitude was significantly more negative for full circle stimuli compared to quarter circle stimuli, particularly at occipital electrodes and under low to moderate noise levels (SN0.0 and SN0.4). This suggests that the completeness of the symmetrical contour enhances object recognition processes, but this advantage is attenuated under high noise conditions. Additionally, the significant interactions between hemisphere, stimulus type, and noise level indicate that the two hemispheres may process symmetrical patterns differently, with the right hemisphere showing greater sensitivity to noise.

The P260 component, peaking around 260 ms, is associated with late-stage perceptual decision-making and response selection (Luck and Hillyard, 2010). Our results revealed that the P260 amplitude was less positive for full circle stimuli compared to quarter circle stimuli under moderate noise levels (SN0.4), particularly at parietal electrodes. This suggests that the completeness of the symmetrical contour facilitates decision-making processes, but this effect is modulated by noise. Furthermore, the significant differences in P260 amplitude across all noise levels indicate that noise affects not only early sensory processing but also later stages of perceptual decision-making.

### 4.1 Hemispheric specialization

One of the key questions addressed in this study was whether there is hemispheric specialization for the detection of reflection symmetry. Our ERP results provide partial support for this idea, with significant hemisphere effects observed at multiple stages of processing. For example, the P/N100 component showed more negative amplitudes in the left hemisphere at parietal electrodes, while the N190 and P260 components revealed significant interactions between hemisphere, stimulus type, and noise level. These findings suggest that the two hemispheres may play distinct roles in symmetry perception, with the left hemisphere potentially specializing in the processing of local features and the right hemisphere in the integration of global shapes (Fink et al., 1997; Sasaki et al., 2005). However, further research is needed to clarify the specific contributions of each hemisphere to symmetry perception.

### 4.2 Implications for the callosal hypothesis

The callosal hypothesis posits that the anatomical symmetry of the human visual system, particularly the role of the corpus callosum in interhemispheric communication, underlies the efficiency of vertical symmetry detection (Wright, 1972). While our findings do not directly contradict this hypothesis, they suggest that symmetry perception involves more complex interactions between the two hemispheres than previously thought. For example, the significant hemisphere effects observed in our study indicate that symmetry processing is not solely determined by the anatomical midline but is also influenced by functional specialization and task demands. This aligns with recent reviews suggesting that the strong version of the callosal hypothesis is unlikely to fully account for the observed phenomena in symmetry perception (Bertamini et al., 2018; Treder, 2010).

## 5 Conclusion

In conclusion, our study provides new insights into the neural and cognitive mechanisms underlying symmetry perception. The behavioral and ERP results demonstrate that noise disrupts contour integration and perceptual grouping, with significant effects observed at multiple stages of processing. Additionally, the findings suggest that the two hemispheres may play distinct roles in symmetry perception, with the left hemisphere potentially specializing in local feature processing and the right hemisphere in global shape integration. These results challenge the strong version of the callosal hypothesis and highlight the need for a more nuanced understanding of the factors influencing symmetry perception. Future research should continue to explore the interplay between neural, cognitive, and ecological factors in shaping our perception of symmetry.

## Data Availability

The raw data supporting the conclusions of this article will be made available by the authors, without undue reservation.
